# Lipidomic Signatures for Colorectal Cancer Diagnosis and Progression Using UPLC-QTOF-ESI^+^MS

**DOI:** 10.3390/biom11030417

**Published:** 2021-03-11

**Authors:** Claudiu Răchieriu, Dan Tudor Eniu, Emil Moiş, Florin Graur, Carmen Socaciu, Mihai Adrian Socaciu, Nadim Al Hajjar

**Affiliations:** 1 Surgery Department, County Hospital Alba, 510118 Alba Iulia, Romania; crachieriu@yahoo.com; 2 Iuliu Hatieganu University of Medicine and Pharmacy, Regional Institute of Gastroenterology and Hepatology “Octavian Fodor”, 400015 Cluj-Napoca, Romania; drmoisemil@gmail.com (E.M.); graurf@yahoo.com (F.G.); na_hajjar@yahoo.com (N.A.H.); 3 Oncology Department, Iuliu Hațieganu University of Medicine and Pharmacy, 400015 Cluj-Napoca, Romania; tudor.eniu@umfcluj.ro; 4 University of Agricultural Sciences and Veterinary Medicine, 400372 Cluj-Napoca, Romania; 5 Research Center for Applied Biotechnology in Diagnosis and Molecular Therapy, 400478 Cluj-Napoca, Romania

**Keywords:** metabolomics, biomolecules, putative biomarkers, colorectal cancer, high-performance liquid chromatography, mass spectrometry, Metaboanalyst

## Abstract

Metabolomics coupled with bioinformatics may identify relevant biomolecules such as putative biomarkers of specific metabolic pathways related to colorectal diagnosis, classification and prognosis. This study performed an integrated metabolomic profiling of blood serum from 25 colorectal cancer (CRC) cases previously classified (Stage I to IV) compared with 16 controls (disease-free, non-CRC patients), using high-performance liquid chromatography and mass spectrometry (UPLC-QTOF-ESI^+^ MS). More than 400 metabolites were separated and identified, then all data were processed by the advanced Metaboanalyst 5.0 online software, using multi- and univariate analysis, including specificity/sensitivity relationships (area under the curve (AUC) values), enrichment and pathway analysis, identifying the specific pathways affected by cancer progression in the different stages. Several sub-classes of lipids including phosphatidylglycerols (phosphatidylcholines (PCs), phosphatidylethanolamines (PEs) and PAs), fatty acids and sterol esters as well as ceramides confirmed the “lipogenic phenotype” specific to CRC development, namely the upregulated lipogenesis associated with tumor progression. Both multivariate and univariate bioinformatics confirmed the relevance of some putative lipid biomarkers to be responsible for the altered metabolic pathways in colorectal cancer.

## 1. Introduction

Colorectal cancer (CRC) is an important public health issue, among the three leading causes of cancer-related mortality in both men and women, according to recent cancer statistics [1,2,3,4], particularly in Western countries but also in developing countries, and is strongly related to lifestyle, stress, food diet and habits.

The early detection and endoscopic resection of adenomatous polyps (premalignant conditions) and screening colonoscopy significantly improve the survival rate, being considered a gold standard for the detection of colorectal neoplasms, beside sigmoidoscopy, colon capsule endoscopy and magnetic resonance colonography. The biopsy specimens of colorectal mucosa and colonic lesions are also useful diagnosis procedures; however, all these techniques are invasive. This is the reason why scientists are more and more keen on using non-invasive techniques with good predictive value and high sensitivity such as metabolomics. As reviewed recently, the management of colorectal cancer was changed radically, using omics technologies for finding diagnosis, stratification and prognosis biomarkers, as well as for treatment monitoring [5].

Metabolomics-based procedures using biofluids (especially blood serum or plasma) assure a systematic screening or fingerprinting of small metabolites (with less than 2000 Da) related to the metabolic signature and pathway alterations in different stages of CRC. Metabolomics and metabonomics offer a qualitative untargeted signature (fingerprint) or a targeted methodology with quantitative evaluation of putative biomarkers [6,7,8,9,10,11].

Metabolomic investigations include mainly gas chromatography or high-performance liquid chromatography coupled with mass spectrometry (GC-MS, HPLC-MS) and magnetic resonance (NMR). By far the most applied technique is based on LC-MS, as it has superior detection and identification capability [12,13,14,15]. Recently, the serum fatty acid profiling of colorectal cancer was reported, using either gas chromatography – mass spectrometry [16,17], NMR [18] or Fourier transform ion cyclotron resonance mass spectrometry [19].

Different metabolic alterations are associated with colorectal cancer (CRC), since cancer cells are able to generate energy even in a nutrient-deficient environment and prefer glycolysis against oxidative phosphorylation as demonstrated for years (the Warburg effect). However, recently this paradigm shifted towards a “reversed Warburg effect”: some cancer cell types, including CRC cells, may synthesize ATP by mitochondrial phosphorylation [20], realizing metabolic remodeling and alterations of mitochondrial respiration [21], opening new research directions for the identification of molecular therapeutic targets, such as fatty acid (FA) synthesis and oxidation.

The metabolization of exogenous glutamine represents another dependence of cancer cell, with many oncogenic mutations affecting glutamine metabolism [22]. Meanwhile, alterations of lipid metabolism in CRC lead to structural changes in cell membranes and disruption of energy homeostasis, cell signaling, gene expression and protein distribution, affecting a number of cell functions, such as proliferation, differentiation, apoptosis, autophagy, necrosis, and drug and chemotherapy resistance [23].

The growing interest related to the role of lipids and their metabolism in cancer development has been presented in previous reviews [1,24,25]. The lipid metabolic pathways are affected in CRC cells and include FA synthesis, desaturation, elongation and mitochondrial oxidation. A plasma lipidomic signature reveals perturbed lipid metabolic pathways and potential lipid biomarkers of human colorectal cancer [26]. The complex lipid metabolic changes may be explained by the high proliferation rate of the CRC cells, with high energetic needs and changes in the serum levels of phospholipid components derived from cell membrane degradation, accompanied by inflammation and changes in the arachidonic acid metabolites in serum or tissue. Recently, an integrated multi-omics approach and lipidomic-based characterization of the lipid metabolism in colorectal cancer were reported [27,28,29,30].

The explanation for increased levels of choline-related metabolites in tumors is probably the result of the accelerated lipid membrane metabolism involved in ATP generation, due to rapid cell proliferation. Glucose changes were consecutive to glycolysis and upregulated in CRC, while increases in 3-hydroxybutyrate, an end metabolite of fatty acids, suggested that the upregulation of fatty acid β-oxidation needed as energy support for cancer cell proliferation [31]. Increased oxidative stress is usually associated with increased oxidation of fatty acids, which may result in an accumulation of 3-hydroxybutyrate [32]. The predictive value of lipid biomarkers is very important, the main predictive factor being considered the stage of diagnosis [4], which explains the importance of CRC screening and early diagnosis.

Different prediction models for CRC patients compared with controls included metabolites such as 2-hydroxybutyrate, aspartic acid, kynurenine and cysteamine [33], or pyruvic, fumaric and glycolic acids; palmitoleic acid; ornithine; lysine tryptophan and 3-hydroxyisovaleric acid [17]. When serum fingerprints from CRC patients were compared prior to surgery and one month after, the potential biomarkers belonged to lipid classes (phosphatidylcholines (PCs), lysophosphatidylcholines (LPCs) and diacylglycerols (DGs)), without a significant difference between the pre-operative and post-operative status [15]. For the assessment of the CRC recurrence rate and survival of the patients after surgical intervention or chemotherapy, 15 metabolites, including four lipids (glycerol, myristate, palmitoleate and 2-aminobutyrate), were selected as potential biomarkers [32].

Recently, a relevant serum MS study for lipophilic metabolites performed under the European Prospective Investigation into Cancer and Nutrition (EPIC), reported nine metabolites to be related to CRC etiology and were recommended for further CRC prospective studies [34]. It was concluded that changes in plasma lipid composition preceded the appearance of neoplasia and that tumor changes can induce a global change in LPC metabolism [35]. Another prospective study found 35 metabolites associated with CRC risk, including 12 glycerophospholipids with an important role in the risk of developing colorectal cancer [36].

Our previous reports focused on the selection of some lipidomic biomarkers to diagnose CRC, based on literature surveys [37,38]. In this context, this experimental study aimed at the identification of specific blood serum biomarkers from patients diagnosed with CRC in four progression stages. The UPLC-QTOF-ESI^+^ MS results, combined with a succession of multivariate and univariate statistical models, including ANOVA, partial least squares discriminant analysis (PLSDA), cluster analysis, random forest and pathway analysis, showed the predictive value of specific biomolecules to be considered as putative CRC biomarkers.

## 2. Materials and Methods

### 2.1. Patients and Compliance with Ethical Standards

The protocol of this study was approved by the Ethics Committee of the Cluj-Napoca “Iuliu Hatieganu” University of Medicine and Pharmacy, including collection of the details about samples and the individual, and the written consent of all subjects, before entering them in the study. The CRC patient group (25 patients) included 16 men and 9 women operated on in the Surgery Department, Regional Institute of Gastroenterology and Hepatology “Octavian Fodor” Cluj-Napoca, Romania, between May and December 2018, with confirmed CRC, either before surgery (suspicion through colonoscopy) or post-surgery (having preoperative radiological suspicion of CRC). The clinical and pathological features as well the stage of CRC tumor were established according to histological evaluation and pTNM classification, as presented in Table 1.

The control group included 7 males (53.1 ± 6.7 years) and 4 females (56.33 ± 8.98 years) considered to be CRC-free, with a negative colonoscopy for cancer or adenoma during the last 12 months. The controls were operated on in the same hospital for other benign diseases (inguinal or incisional hernia, benign skin lesions, cholelithiasis).

Co-morbidities like morbid obesity, insulin-dependent diabetes mellitus, liver cirrhosis, adenomatous polyps and a history of inflammatory bowel disease were excluded in both groups. The blood samples were collected similarly from all participants, in hospital, prior to surgery, in the morning, after a minimum of 12 h fasting.

In parallel, data about other clinical and preclinical parameters of the patients and controls were registered but not included in this report.

### 2.2. Blood Collection and Processing

Blood serum samples were collected according to standardized procedures in accordance with the ethical standards of the institutional and national research ethical committee and with the 1964 Helsinki Declaration and its later amendments for ethical standards.

The blood was collected in vacutainer tubes without anticoagulant, kept at room temperature for 30 min to allow clotting and centrifuged for 10 min at 3000 rpm (4 °C) to separate clear serum. After separation, the blood serum was stored at −80 °C. To a volume of 0.2 mL serum, 0.8 mL of a mixture of methanol and acetonitrile (1:1) was added to precipitate proteins. The mixture was vortexed for 1 min, kept at −20 °C overnight and then vortexed again for 1 min. After mixing, the vials were centrifuged at 12,500 rpm for 10 min and the supernatant was collected and filtered through PTFE filters of 0.25 µm.

### 2.3. HPLC–ESI(+)-QTOF-MS Analysis of Blood Serum

Aliquots of 3 µL of serum were subjected to ultrahigh-pressure chromatography on a Thermo Scientific HPLC UltiMate 3000 (Waltham, MA, USA) system equipped with a quaternary pump system DionexUltiMate 3000 (UHPLC) (ThermoFischer, Waltham, MA, USA), a DionexUltimate 3000 photodiode array detector, a column oven and autosampler. Serum metabolites were separated using a Thermo Scientific C18 reverse-phase column (Acquity, UPLC C18 BEH, Waters Corporation, Milford, MA, USA) (5 µm, 2.1 × 75 mm) at 25 °C and a flow rate of 0.3 mL/min. The mobile phase was represented by a gradient of Eluent A (water containing 0.1% formic acid) and Eluent B (methanol:acetonitrile, 1:1, containing 0.1% formic acid). The gradient system consisted of 99% A (Minute 0), 70% A (Minute 1), 40% A (Minute 2), 20% A (Minute 6) and 100% B (Minute 9–10), followed by 5 min with 99% A. The total running time was 15 min. The mass spectrometry was performed on a Bruker Daltonics MaXis Impact QTOF (Bremen, Germany) instrument, operating in positive ion mode (ESI+). The mass range was set between 50 and 1000 m/z. For measurements, the nebulizing gas pressure was set at 2.8 bar, the drying gas flow at 12 L/min and the drying gas temperature at 300 °C. Before each chromatographic run, a calibrant solution of sodium formate was injected. The control of the instrument, the acquisition and data processing were done using Chromeleon, TofControl 3.2, Hystar 3.2 and Data Analysis 4.2 (Bruker Daltonics, Bremen, Germany).

### 2.4. Statistical Analysis

The Bruker software attached to the instrument, Data Analysis 4.2, was used to process the acquired data. First, from the total ion chromatogram, using specific algorithms base peak chromatograms were obtained, and the Find Molecular Features (FMF) algorithm generated an advanced bucket matrix. The matrix released by Data Analysis contained the retention time, the peak areas and intensities and the signal/noise (S/N) ratio for each component, together with its m/z value. Generally, the number of separated compounds ranged between 600 and 800.

In this first step, a matrix for all samples was obtained and stored in an Excel file. In order to eliminate the small signals with S/N values under 10, an initial filtration (1) was made and then a second matrix containing m/z values and peak intensities was saved and filtered in a second step eliminating the small intensities (2). Generally, the number of peaks remained at 180–220. Only metabolites which were detected in more than 80% of the samples were included in the statistical analysis, so to make an adequate alignment of the peak m/z values, the online software from bioinformatica.isa.cnr.it/NEAPOLIS was applied. The aligned matrix (3) allowed the calculation of mean Intensity values and standard deviations for the control group and for subgroups corresponding to CRC Stages I–IV. The aligned matrix was then converted to a csv file and introduced in the specialized online software Metaboanalyst 5.0.

After successive alignment and normalization of the matrix data, the multivariate analysis consisted of the representation of fold change, volcano plot, principal component analysis (PCA), partial least squares discriminant analysis (PLSDA) and random forest, finding correlations between samples and between variables (m/z values), as well as building the heatmap which represents the correlation between variables and samples. Finally, using the biomarker analysis, the receiver operating curves (ROCs) were obtained and the values of the areas under the ROC curves (AUCs) were obtained and the molecules identified were ranked according to their sensitivity/specificity. The enrichment analysis and the MS to pathways algorithm allowed the identification of specific alterations of metabolic pathways induced in CRC.

The identification of molecules which can be considered potential biomarkers was made using the 2 most relevant databases, LIPID MAPS Lipidomics Gateway and the Human Metabolome Database.

## 3. Results

### 3.1. Multivariate Analysis

#### 3.1.1. PCA and PLSDA Analysis

**Figure 1:**
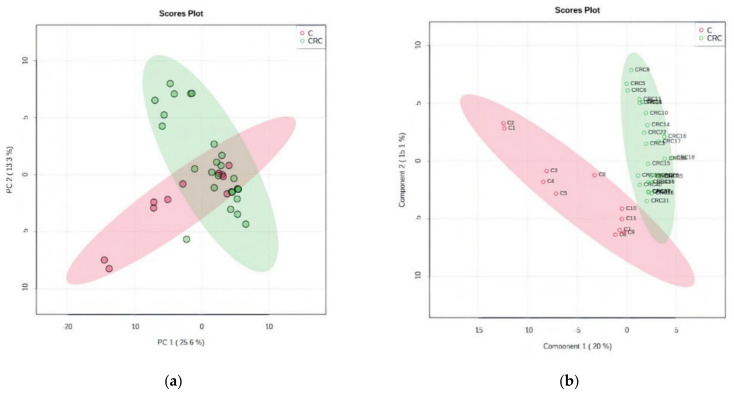
(**a**) Principal component score plot showing the homogeneity and C and CRC groups. (**b**) Partial least squares discriminant analysis (PLSDA) plot with sample identification, showing the discrimination between C and CRC groups.

By unsupervised PCA, the co-variance for the first five components was evaluated. The explained variance in serum groups (CRC and C) was 25.6% (PC1) and 13.3% (PC2), covered by a total variance of 38.9% (Figure 1a). The discrimination between CRC and C groups was better represented by PLSDA (covariance of 35.1%) (Figure 1b).

According to the PCA and PLSDA plots, the C group was less homogeneous than the CRC group, two or three subgroups being visible in this group. This can be explained by the diverse co-morbidities of the patients from this non-CRC group.

The cross-validation algorithm showed a high accuracy (close to 1), high *R*^2^ and significantly high Q2 values, its performance increasing from Component 1 to 3, up to 0.7 (Figure S1a, Supplementary Materials). These data indicated very good validation and predictability for this model.

#### 3.1.2. Euclidean Dendrogram and Correlation Heatmaps

**Figure 2:**
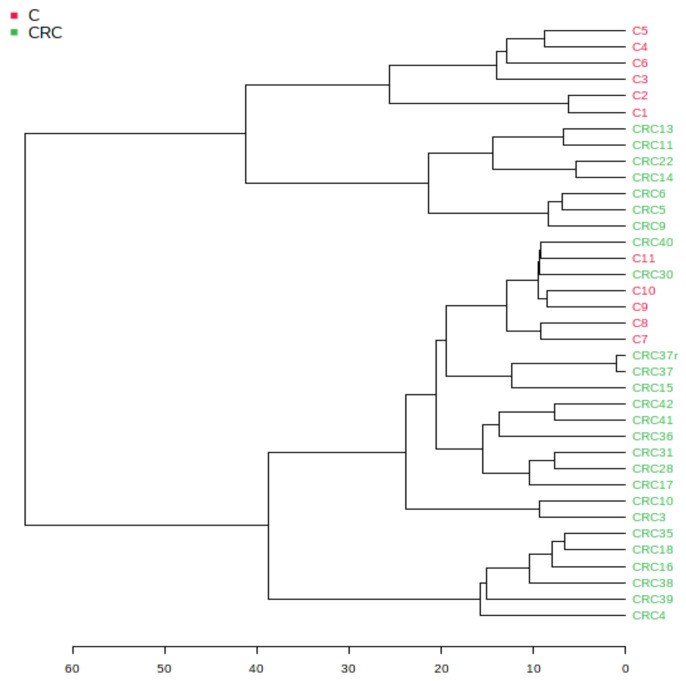
Hierarchical clustering dendrogram of samples using the Euclidian distance measure and the Ward clustering algorithm.

Figure 2 shows the hierarchical clustering of different samples, displayed as a tree diagram called a dendrogram. The hierarchical clustering dendrogram was chosen, with an Euclidian distance measure and the Ward clustering algorithm, one of the options found in Metaboanalyst 5.0. Using the Euclidian algorithm, in the scale of distances from 1–60, one can see good similarities (distances of <35) between the individual samples from the CRC group. The pathologic CRC group (marked in green) showed a good similarity while the group C (marked red) was split into two subgroups.

Figure S2a–c (Supplementary Materials) represents the maps of correlation between variables (m/z values) and between samples, as well the heatmap showing the correlations between samples and variables. These maps were built considering the *t*-test and *p*-values obtained by applying the Metaboanalyst 5.0. algorithm. The colors represent positive (red) or negative (blue) correlations as determined by the *t*-test. One can discriminate different patterns for samples correlated with variables (blue zones differentiated from red zones). In the heatmap (Figure S2c), the red spots show the molecules which have increased levels in certain samples, while blue spots reflect decreases in certain molecules for specific samples.

#### 3.1.3. The Random Forest Algorithm and Its Predictive Value

This algorithm was able to indicate the predictive value (as potential biomarkers) for some molecules which differentiated the CRC and C groups. Table 2 presents the m/z values of the first 30 molecules to be considered as predictive by the random forest algorithm. The MDA values from 0.012 to 0.002 were considered and the decrease (D) or increase (I) in the level of these molecules in the CRC vs. C groups was seen.

#### 3.1.4. Biomarker Analysis

According to Metaboanalyst software, biomarker analysis includes the receiver operating characteristic (ROC) curve as a useful tool to evaluate the diagnostic accuracy. Many biomarker combination methods rely on maximization of the area under the ROC curve (AUC). This parameter allowed the evaluation of the sensitivity versus specificity of each molecule to be considered a relevant biomarker. Higher values of AUC close to 1 for a certain molecule mean higher prediction to be considered as a biomarker. Table 3 shows the m/z value and putative identification, AUC value, *p*-values and log2FC values for each molecule identified, as well its variation in the CRC group vs. C group.

Significantly high AUC values above 0.750 showed that 25 molecules might be considered as putative biomarkers; these molecules belong to different lipid classes.

These data confirm that lipid molecules, mainly choline-dependent phospholipids, ceramides and different esters (of fatty acids or cholesterol), can be considered as predictive molecules with high prognostic values for CRC diagnosis.

### 3.2. Univariate Analysis ANOVA: Discrimination of CRC Stages

#### 3.2.1. One-Way ANOVA to Identify Biomarkers for CRC Progression (Stages I to IV)

**Figure 3:**
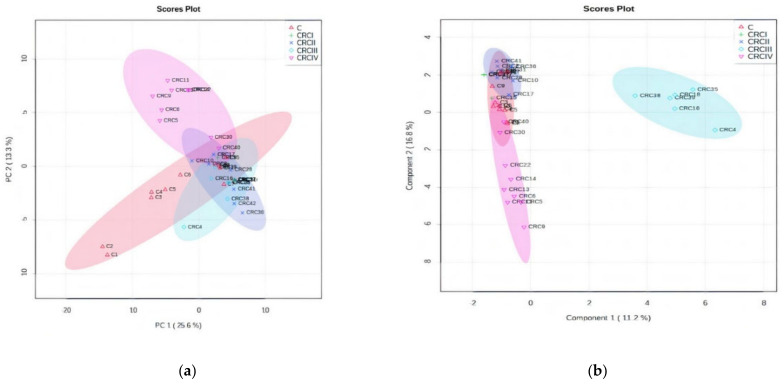

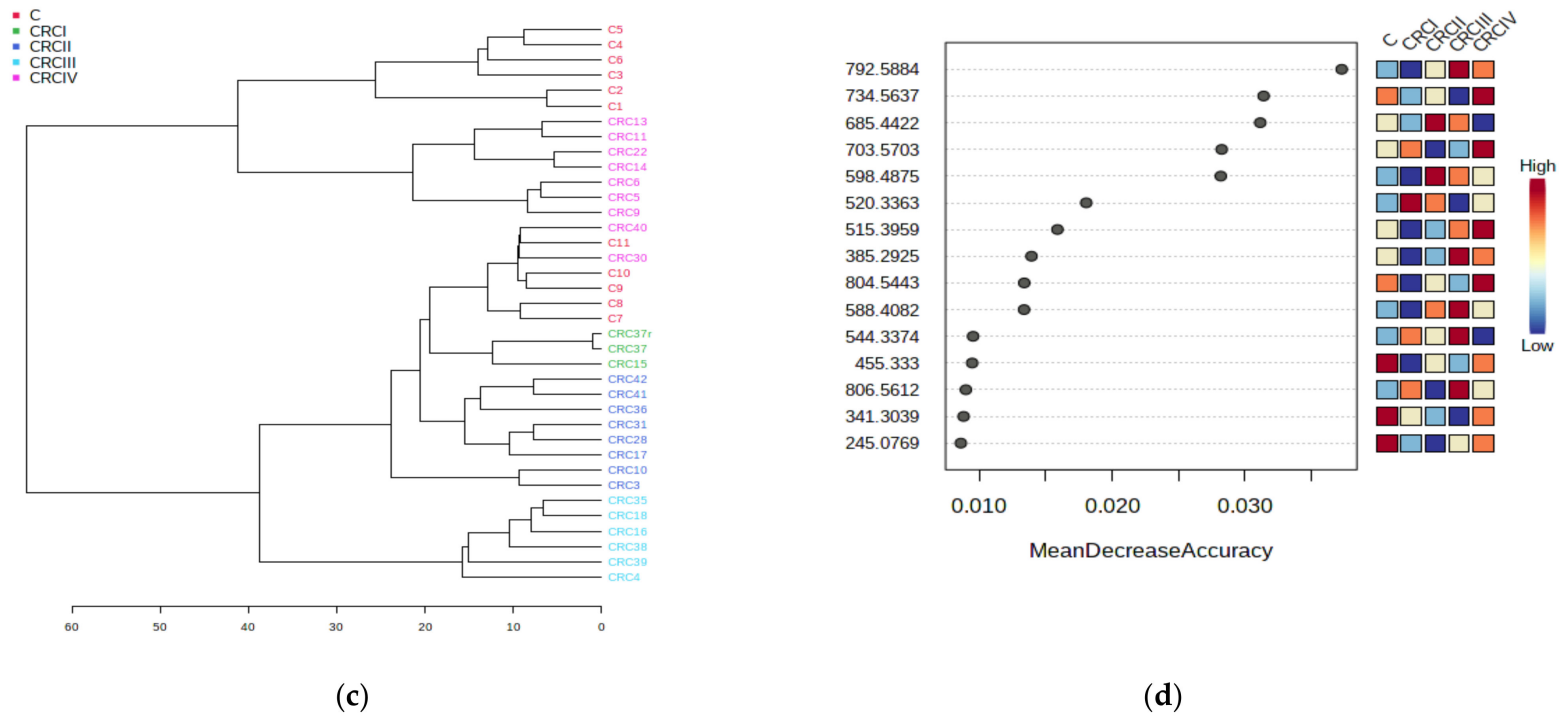
ANOVA of CRC stages. (**a**) Principal component analysis (PCA) score plot (**b**) PLSDA score plot. (**c**) Dendrogram showing the clustering of CRC I–IV and C subgroups. (**d**) The graph of random forest analysis: MDA values for the first 15 predictive molecules.

Applying the ANOVA univariate analysis, included in the Metaboanalyst software, Figure 3a,b presents the PCA and PLSDA plots. The PCA plot showed a covariance of 38.9%, while PLSDA showed a covariance of 28% that was able to discriminate between the C and CRCI–IV groups. In the PCA plot, the subgroups CRCIV and CRCII are well discriminated as well, in the opposite direction compared with the C group and the CRC I and III subgroups. Interestingly, in the PLSDA plot, the CRCIII subgroup had a significant difference from the other subgroups. Finally, considering the inputs from these plots, we consider that CRCIII and CRCIV are the CRC stages to show significant differences to be considered in chemo-statistic evaluations. The cross-validation graphic (Figure S1b) shows the high accuracy and significance of the PLSDA model: an accuracy value close to 1 indicates a very good description of the data by the model, whereas the *R*^2^ and high Q2 values confirm the model’s performance, increasing from Component 1 to 5, up to 0.7.

The dendrogram (Figure 3c) shows the clustering of subgroups CRC I–IV and Figure 3d shows the MDA values of the model, as determined by the random forest algorithm, including the first 15 predictive molecules.

As can be seen in Figure 3a,b, clear delimitation of Stages III and IV were observed either by the PCA or PLSDA score plots. The loading analysis and MDA values showed which are the molecules considered to be responsible for this strong discrimination. Table 4 includes the first 30 molecules and their MDA values (up to 0.002). The increase (I) or decrease (D) of these molecules in the CRC vs. C group the included.

#### 3.2.2. Statistical Analysis Based on MS Peak Intensity Values for CRC Subgroups

In order to compare the data obtained by multivariate analysis, we considered the initial matrices (peak intensity tables), considering the mean values for the CRCI-IV and C groups, and calculated the statistical differences between these groups (values from *p* < 0.1 to *p* < 0.01 indicate significance). From a total of 93 biomolecules (included in Table S1), significant deviations of these ratios (*p* < 0.01) were selected. The data released from LC-MS analysis were presented in a matrix representing the average values of MS peak intensity values for each molecule separated and selected according to the protocol presented above (*n* = 45). Table S1 includes the list of common molecules identified in blood serum from C and CRC groups. The ratios between the mean values of CRCIV and C, CRCIV and I, CRCIV and III, and CRCIII and C are presented in Table 5, as well the tentative identification of the molecules and their codification in the PubChem database.

These data suggest general increases in different lipid subclasses such as choline-dependent glycerophspholipids, cholesterol and fatty acid esters, as well ceramides, especially in Stages III and IV compared with controls. Stearic and palmitic acids are mostly involved in such esters. These data are in good agreement with the multivariate analysis.

#### 3.2.3. Enrichment and Pathway Analysis

Using enrichment analysis and pathway analysis by the GSEA algorithm for the matrix including the m/z values presented in Table 5 and decreasing *p*-values and *t*-scores, the possible pathways affected by CRC in different stages were obtained.

**Figure 4:**
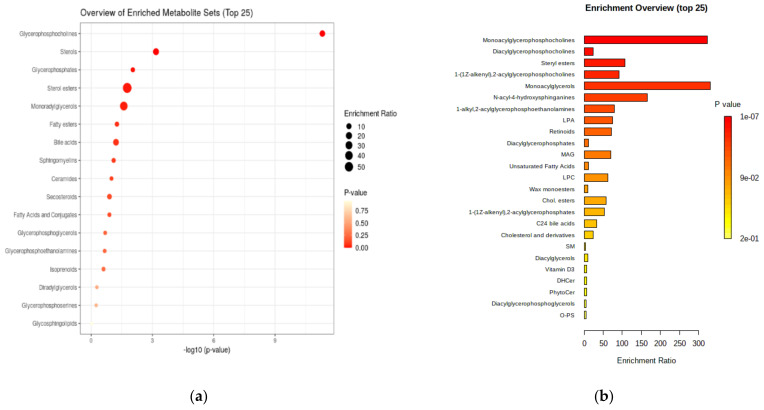
(**a**) General overview of the enriched metabolite class sets (top 17) expressed as log 10 (*p*-value). (**b**) Detailed enrichment overview for molecule subsets and their enrichment ratios.

Figure 4 presents a general and detailed overview of the enriched metabolite classes sets and their significance.

The results plotted above confirm the metabolite sets which can be considered as significant in CRC diagnosis and prognosis, including their classifications. The main class of metabolites is represented by the glycerophosphocholines (mono- and diacyl derivatives), followed by sterols and their esters, ceramides and sphingomyelins.

## 4. Discussion

The results of this study may contribute to the actual knowledge directed towards identification of the most relevant biomarkers of CRC and progression stage subtypes, compared with controls. These data are mostly in good agreement with previous findings which reflect the key role of lipid-mediated pathways in CRC diagnosis and prognosis.

As reported before, choline-related phospholipids can be considered good biomarkers for CRC [39,40]. Increases in PCs may be followed by decreases in LPCs, associated with body weight loss and activated inflammatory processes in CRC patients [41] but also by accumulation of some LPCs (16:0, 16:1 and 18:0) [42] oran increased degradation rate of some LPCs (20:4 and 22:6) as a result of the accelerated cell proliferation in CRC patients [36,43]. Another lipid metabolic signature represented by palmitic amide, oleamide, octadecanoic, hexadecanedioic, myristic and eicosatrienoic acids, LPCs(16:0, 18:2, 20:4, 22:6) was statistically significant and these lipid metabolites were considered potential biomarkers to discriminate early-stage patients from healthy controls, superior to the prediction made by carcinoembryonic antigen [40]. Endogenous synthesis of arachidonic and oleic acids was also reported to have an impact on CRC development, as well as the arachidonic acid metabolites (eicosanoids and their oxidized forms), which generate prostaglandin E2 which stimulates tumorigenesis [44]. Meanwhile, no significant differences between normal, polyp and cancer mucosa were noticed for oxidized lipids 12-hydroxyeicosatetraenoic acid (HETE), 15-HETE or leukotriene B4 levels, or decreased 13-hydroxyoctadecadienoic acid (HODE) and HETE levels in cancer and colorectal polyp mucosa [15,27,45]. The upregulated and downregulated metabolites through the various stages of CRC were found to be also benzoic, octanoic and decanoic acids, proportional to CRC stage [35,46,47]. Glyceraldehyde, hippuric and linolenic acids, glycochenodeoxycholate and glycocholate may also discriminate CRC from polyps [12], while β-hydroxibutyrate increased and tryptophan and indoleacrylic acid decreased from Stage I to Stage IV CRC [48,49,50]. By comparison, the healthy, polyp adenomas and CRC patients had different glycerolipid metabolism, reflected by higher levels of lipids and polyunsaturated fatty acids (PUFAs) and lower levels of glycerol [18].

In plasma, previous studies reported the upregulation of fatty acid synthesis, increased LPCs (26:0 and 28:0) [47,50] and other types of LPC [19,27,39], increased Monounsaturated fatty acid (MUFA)/PUFA ratios [51], and increased ethanolamine plasmalogens, polyunsaturated fatty acids and polar lipids [26,27,51,52]. In serum, there were noticeable increases in triglycerides containing C15:0,18:0, 18:1,18:2 and 18:3 [53,54] or decreases in lipids with C22:0, 24:0, 26:0, 30:0 and 18:1 [16]; and decreases in ultralong chain fatty acids [34]; increases in endocannabinoids and ceramides, sphingomyelins, eicosanoids [8,31,43,46], succinate, dimetihylguanosine, adenine, citraconic acid and methylguanosine [40]. Cerotic acid may also be a novel serum metabolic marker of colorectal malignancies [55], as well as plasma triacylglycerols [56,57].

To summarize the findings presented above, in relation to our results we can assume that in CRC, several classes of lipids including phosphatidylglycerols (PCs, phosphatidylethanolamines (PEs) and phosphatidic acids (PAs)), fatty acids and sterol esters, as well as ceramides, confirm the “lipogenic phenotype” for CRC development, dependent on lipogenesis and lipolysis, upregulated and associated with tumor progression. Both multivariate and univariate bioinformatics confirm these findings and the specificity of these metabolic pathways activated in CRC patients [58].

Further studies are under development using larger cohorts of patients in different CRC stages, with improved characterization and data processing.

## 5. Conclusions

Metabolomics has already proven a great potential as a high-value technology to realize proper metabolic signatures to discriminate significantly between healthy controls with benign polyps versus malignant CRC tumors. Specific classes of lipids involved in cellular signaling and energy provision proved to be good biomarkers for CRC in different stages and can be relevant prognosis factors. The lipid profile alterations presented in this study, many of them also confirmed by similar investigations, showed statistically significant differences and can be considered reliable biomarkers, differentiating between early and advanced stages of this malignancy, or serving as survival predictors. Complementary studies on larger cohorts of patients are needed for the development of clinically useful biomarkers, especially related to the signaling lipids.

Metabolomics have the potential to become a standard technology for future applications in translational cancer research, but further, large-scale studies and prospective validation are still needed. Moreover, bioinformatics tools offered by the online Metaboanalyst 5.0 software significantly helped refining of the key biomolecules which may be considered as putative biomarkers for CRC diagnosis and staging. These biomarkers are not only useful for diagnostics and patient stratification but can be mapped on a biochemical chart to identify the altered metabolic pathways involved in the initiation and progression of this invasive cancer.

## Figures and Tables

**Table 1:** Clinical pathological features of the colorectal cancer (CRC) patients included in this study.

Biospecimen	CRC	Tumor Site	Co-Morbidities
Number of participants	25	-	-
Male Age (mean ± SD) Female Age (mean ± SD)	64.12 ± 13.94 69.11 ± 9.34	-	-
Male/female Nr	16/9	-	-
Body mass index Male/female	32.5 ± 4.7 25.5 ± 6.7	-	-
pT2NoMoLoVo, Stage I	2 (8%)	Rectosigmoid/rectum	Obesity
pT3NoMoLoVo, Stage-IIA	8 (32%)	Left/right colon	Obesity, IHD, Aortic stenosis, Hemorrhoids
pT3/4aNoMoLoVo, Stage-IIIB	5 (20%)	Rectosigmoid/rectum/colon	NIDDM, obesity, IHD
pT4aNoMoLoVo, Stage-IIIC	1 (4%)	Rectosigmoid	Obesity, HTN, NIDDM
pT3/4aNoMoLoVo, TNM Stage-IV	9 (36%)	Sigmoid/rectum/colon and metastasis	Obesity, HTN, NIDDM, Hemorrhoids

Abbreviations: NIDDM, non-insulin dependent diabetes mellitus; HTN, hypertension; PV, portal vein; IHD, ischemic heart disease.

**Table 2:** The m/z values of the first 30 molecules to be considered a predictive by the random forest algorithm. The MDA values from 0.012 to 0.002 were considered and a decrease (D) or increase (I) in the level of each molecule in the CRC vs. C groups.

m/z	MDA	CRC vs. C	m/z	MDA	CRC vs. C
723.5055	0.011848	D	377.1835	0.004234	D
792.5884	0.010493	I	381.2972	0.004234	D
598.4875	0.007986	I	579.2966	0.004096	D
524.37	0.007733	D	359.3152	0.003745	D
341.3039	0.007204	D	804.5443	0.003715	D
391.2841	0.006768	D	529.3726	0.002683	D
455.333	0.006587	D	707.486	0.002601	D
520.3363	0.006484	I	611.3532	0.002516	D
679.4944	0.00603	D	703.5703	0.002481	D
588.4082	0.005828	I	758.5642	0.00238	D
751.5213	0.004866	I	830.5572	0.002307	D
794.5973	0.004584	D	685.4422	0.002176	D
722.5123	0.004329	D	473.3446	0.002169	D
808.5757	0.004291	D	683.43	0.002123	D
628.46	0.004263	D	782.5624	0.002123	D

The identification of these molecules is presented in Table 3 and Table 4.

**Table 3:** The m/z, area under the curve (AUC), *p*-values and log2FC values, and identification of molecules, based on HMDB and Lipidmaps. Variation in the PS group versus the CS group: I, increase; D, decrease. Identified only blood.

m/z	Tentative Identification	AUC	*p*-Value	Log2FC	CRC vs. C
598.4875	Cer(t18:0/19:0)	0.94056	2.0072 × 10^−4^	−1.0491	I
792.5884	PC(P-18:0/20:5)	0.88811	0.00741	−2.199	I
760.578	PC(18:1/16:0)	0.88462	3.498 × 10^−4^	−0.84091	I
533.2813	Linoleyl stearate	0.84615	9.6101 × 10^−4^	−0.32704	I
642.5126	GlcCer(d14:2/16:0)	0.83566	0.0051776	−0.50237	I
509.4034	Stearyl palmitate	0.83217	0.0015194	−0.30749	I
758.5642	PC(18:1(11Z)/16:1(9Z))	0.82867	0.010393	−0.8229	I
675.54	20:3 Cholesterol ester	0.81818	0.099092	−0.68236	I
551.3605	Retinol oleate	0.81119	0.0018414	−0.92325	I
520.3363	PC(18:2(9Z,12Z)/0:0)	0.8042	0.032773	−1.7684	I
732.5489	PC(16:0/16:1)	0.7972	0.063087	−0.74595	I
341.3039	9-Hexadecenoylcholine	0.78671	0.0049616	0.84315	D
485.3469	PA(22:5(7Z,10Z,13Z,16Z,19Z)/0:0)	0.78322	0.026174	−0.42322	I
515.3959	PA (24:4/0:0)	0.78322	0.010955	−0.8075	I
588.4082	Cer(d18:3/20:1)	0.77273	0.010821	−1.0561	I
716.5108	PE(18:2/16:0)	0.76923	0.10133	−0.33135	I
808.5757	PC(18:0/20:5(5Z,8Z,11Z,14Z,17Z))	0.76573	0.011384	0.35853	I
663.4599	PG(14:1/14:1)	0.76224	0.053647	−1.1222	I
679.4944	20:1 Cholesterol ester	0.76224	0.43243	0.85535	D
359.3152	Tetracosapentaenoic acid (24:5n-3)	0.75874	0.019955	0.79973	D
597.4554	DG(16:0/18:0/0:0)	0.75874	0.0029561	−0.45245	I
814.5707	PC(18:0/20:2(5Z,11Z))	0.75175	0.068745	−1.2218	I
355.2819	MG(18:2(9Z,12Z)/0:0/0:0)[rac]	0.75175	0.042076	−0.39775	I
498.3996	Cer(d18:0/13:0)	0.75175	0.053514	−0.16722	I
703.5703	22:3Cholesterol ester	0.75175	0.017635	−0.60197	I

**Table 4:** The m/z values of the first 30 molecules to be considered predictive by the random forest algorithm. The MDA values from 0.037 to 0.004 were considered, and the decrease (D) or increase (I) in the level of each molecule in the CRC vs. C groups is shown.

m/z	Tentative Identification	MDA	m/z	Tentative Identification	MDA
792.5884	PC(P-18:0/20:5)	0.037295	828.5433	PC(22:6/18:3)	0.007843
734.5637	PE(O-18:0/18:0)	0.031412	732.5489	PE(O-18:0/18:1(9Z))	0.007805
685.4422	PA(P-18:0/18:2)	0.03116	732.5489	PE(O-18:0/18:1(9Z))	0.007805
703.5703	CE(22:3)	0.028253	524.37	PC(18:0/0:0)	0.007498
598.4875	Cer(t18:0/19:0)	0.028186	429.3186	Cholesteryl acetate	0.007431
520.3363	PC(18:2(9Z,12Z)/0:0)	0.018047	701.4414	PA(18:2/18:0)	0.007245
515.3959	PA (24:4/0:0)	0.015878	760.578	PC(18:1/16:0)	0.006782
385.2925	22-dehydrocholesterol	0.013922	780.5458	PC(18:2/18:3)	0.006763
804.5443	PC(18:2/20:5)	0.013375	512.4243	Cer(d16:0/16:0)	0.00673
588.4082	Cer(d18:3/20:1)	0.013362	794.5973	PC(P-18:0/20:4	0.005945
544.3374	PC(20:4/0:0)	0.009519	267.2647	Norlinoleic acid	0.004452
455.333	Vitamin D3 butyrate	0.009452	533.2813	Stearyl palmitate	0.004352
806.5612	PC(18:1/20:5)	0.008977	808.5757	PC(18:0/20:5 (5Z,8Z,11Z,14Z,17Z))	0.00432
341.3039	9-Hexadecenoylcholine	0.008805	642.5126	GlcCer(d14:2/16:0)	0.004228
245.0769	Uridine	0.008599	723.5055	PG(16:0/16:0)	0.004146
828.5433	PC(22:6/18:3	0.007843	707.486	CE(22:1)	0.004095

**Table 5:** M/z values and tentative identification of molecules which show different ratios between the mean values of CRCIV and C, CRCIV and I, CRCIV and III, and CRCIII and C. The codification in the PubChem database is included for each molecule. Significant increases (*p* < 0.01) in these ratios are marked with * symbol.

m/z	CRCIV/C	CRCIV/I	CRCIV/III	CRCIII/C	Tentative Identification	PubChem
267.265	0.987	1.534 *	1.393 *	0.708	Norlinoleic acid	13932174
341.304	0.715	1.079	1.635 *	0.437	9-Hexadecenoylcholine	22155839
355.282	0.957	0.715	0.556	1.721 *	MG(18:2(9Z,12Z)/0:0/0:0)[rac]	5283469
359.315	0.755	3.134	1.260	0.599	Tetracosapentaenoic acid (24:5n-3)	52921801
385.293	1.114	0.976	0.232	4.808 *	22-Dehydrocholesterol	5283661
391.284	0.826	0.814	0.660	1.253	12-Ketolithocholic acid	3080612
455.333	0.813	0.632	0.756	1.075	Vitamin D3 butyrate	14260146
485.347	1.393 *	0.933	0.829	1.680 *	PA(22:5(7Z,10Z,13Z,16Z,19Z)/0:0)	25099711
498.400	1.122	0.700	1.267	0.885	Cer(d18:0/13:0)	52931113
509.403	1.303 *	0.908	0.986	1.322 *	Stearyl palmitate	75778
515.396	2.401 *	3.506 *	0.768	3.124 *	PA (24:4/0:0)	138233301
520.336	2.183 *	0.087	3.290 *	0.664	PC(18:2(9Z,12Z)/0:0)	11005824
522.354	1.440	0.654	1.296	1.111	PC(18:1(9Z)/0:0)	16081932
524.370	0.341	0.293	0.509	0.670	PC(18:0/0:0)	497299
533.281	1.420 *	1.346 *	1.061	1.338	Stearyl palmitate	75778
544.337	0.243	0.104	0.072	3.376 *	PC(20:4(5Z,8Z,11Z,14Z)/0:0)	24779476
551.361	1.787 *	0.792	1.067	1.675 *	Retinol oleate	11699609
588.408	1.227	3.575 *	0.198	6.202 *	Cer(d18:3/20:1)	70678688
597.455	1.295	0.785	0.857	1.512 *	DG(16:0/18:0/0:0)	9543688
598.488	1.365 *	1.022	0.567	2.405 *	Cer(t18:0/19:0)	5322154
628.460	0.407	0.319	0.377	1.080	Cer(t18:0/20:0(2OH))	70678864
642.513	1.142	0.829	0.489	2.337 *	GlcCer(d14:2(4E,6E)/16:0)	70699233
663.460	1.107	0.424	0.522	2.122	PA(16:0/17:0)	52929500
675.540	1.532 *	0.897	1.225	1.251	SM(d16:1/16:0)	52931133
679.494	0.401	1.231	1.184	0.339	20:1 Cholesterol ester	16061337
685.442	0.704	0.522	0.200	3.523*	PA(P-18:0/18:2(9Z,12Z))	52929695
701.441	1.637 *	1.541 *	2.614 *	0.626	PA(18:2(9Z,12Z)/18:0)	52929468
701.530	1.006	2.091 *	5.920 *	0.170	SM(d18:1/16:1)	52931145
703.570	1.851 *	0.648	1.400	1.322 *	22:3 Cholesterol ester	70699301
707.486	0.367	0.292	0.368	0.997	22:1 Cholesterol ester	16219158
716.511	0.981	0.607	0.732	1.341 *	PC(P-16:0/16:1(9Z))	52923882
722.512	1.042	1.157	0.881	1.184	PS(O-16:0/16:0)	52926171
723.506	0.569	0.973	0.676	0.841	PG(16:0/16:0)	446440
732.549	1.949 *	2.911 *	0.994	1.961 *	PE(O-18:0/18:1(9Z))	52924982
734.564	2.722 *	3.062 *	4.165 *	0.654	PE(O-18:0/18:0)	9547051
751.521	1.567 *	0.790	0.971	1.613 *	PG(18:0/16:0)	52927153
758.564	1.156	0.544	0.512	2.258 *	PC(18:1(11Z)/16:1(9Z))	53478719
760.578	2.368 *	1.818 *	1.314 *	1.802 *	PC(18:1(11Z)/16:0)	53478717
792.588	3.593 *	5.039 *	0.280	4.850 *	PC(P-18:0/20:5(5Z,8Z,11Z,14Z,17Z))	52923964
794.597	0.740	0.428	0.557	1.329 *	PC(P-18:0/20:4(5Z,8Z,11Z,14Z))	24779390
804.544	1.052	5.931 *	3.524 *	0.299	PC(18:2(9Z,12Z)/20:5(5Z,8Z,11Z,14Z,17Z))	52922747
806.561	1.303 *	0.480	0.412	3.160 *	PC(18:1(9Z)/20:5(5Z,8Z,11Z,14Z,17Z))	24778949
808.576	0.698	0.672	0.799	0.873	PC(18:0/20:5(5Z,8Z,11Z,14Z,17Z))	24778860
814.571	1.030	0.236	0.818	1.260	PC(18:0/20:2(5Z,11Z))	24778848

## Supplementary Materials

The following are available online at https://www.mdpi.com/2218-273X/11/3/417/s1, Table S1. Matrix representing the m/z values of molecules separated by LC-MS (column 1) and the mean values of mass spectra peak intensity for the C group (column 2) and CRC subgroups representing Stages I, II, III and IV (Columns 3–6). Figure S1. (a) Cross-validation of PLSDA analysis for the CRC and C groups. For interpretation, see the main text. (b) Cross-validation of PLSDA analysis for CRC I to IV subgroups. For interpretation, see the manuscript. Figure S2. Correlation maps between variables (m/z values) (a) and samples (b). The heatmap (c) represents the correlation between samples and variables. All maps were built using the Metaboanalyst 5.0 algorithm (multivariate statistics and *t*-test).

## Abbreviations

Cer	ceramide
CRC	colorectal cancer
DG	diacylglycerol
FFA	free fatty acid
GlcCer	glucosylceramide
HETE	hydroxyeicosatetraenoic acid
HODE	13-hydroxyoctadecadienoic acid
LA	linoleic acid
LPA	lysophosphatidic acid
LPC	lysophosphatidylcholine
MG	monoacylglycerol
MUFA	monounsaturated fatty acid
PA	phosphatidic acid
PC	phosphatidylcholine
PE	phosphatidylethanolamine
PL	phospholipid
PUFA	polyunsaturated fatty acid
SM	sphingomyelin
SPL	sphingolipid
TG	triacylglycerol.
